# Genetic Subtype‐Based International Prognostic Index Prognostic Model in Diffuse Large B‐Cell Lymphoma

**DOI:** 10.1002/mco2.70190

**Published:** 2025-06-16

**Authors:** Lan Mi, Jili Deng, Jiayue Qin, Chen Zhang, Lixia Liu, Shunli Yang, Libin Chen, Hua‐Jun Wu, Haojie Wang, Jun Zhu, Hong Chen, Feng Lou, Shanbo Cao, Yuqin Song, Weiping Liu

**Affiliations:** ^1^ Key Laboratory of Carcinogenesis and Translational Research (Ministry of Education) Department of Lymphoma Peking University Cancer Hospital & Institute Beijing China; ^2^ Department of Medical Oncology Sichuan Clinical Research Center for Cancer Sichuan Cancer Hospital & Institute, Sichuan Cancer Center, University of Electronic Science and Technology of China Chengdu China; ^3^ Department of Medical Affairs Acornmed Biotechnology Co., Ltd. Beijing China; ^4^ Key laboratory of Carcinogenesis and Translational Research (Ministry of Education/Beijing) Peking University Cancer Hospital & Institute Beijing China; ^5^ Center for Precision Medicine Multi‐Omics Research Institute of Advanced Clinical Medicine Peking University Beijing China; ^6^ Department of Biomedical Informatics School of Basic Medical Sciences Peking University Health Science Center Beijing China; ^7^ School of Basic Medical Sciences Center for Precision Medicine Multi‐Omics Research Peking University Health Science Center Beijing China

**Keywords:** diffuse large B‐cell lymphoma, defined genetic subtype, LymphType, International Prognostic Index, integrated prognostic model

## Abstract

Molecular subtyping in diffuse large B‐cell lymphoma (DLBCL) leads to facilitating drug selection. However, an integrated prognostic model based on molecular subtyping and clinical features has not been well established. Here, we retrospectively performed whole genome sequencing, whole exome sequencing, and fluorescence in situ hybridization in newly diagnosed DLBCLs, established a simplified LymphType algorithm for classification evaluation, and proposed a new integrated prognostic stratification system, combined molecular subtypes and International Prognostic Index (IPI) scoring system in our in‐house sequencing cohort (*N* = 100), and validated in three public cohorts (*N* = 1480). Compared with IPI scoring system and classification algorithm model alone, the discrimination ability of prognostic model based on the new integrated model showed best discrimination of overall survival with concordance index value (0.773 vs. 0.724 vs. 0.648). We subsequently established a four‐category risk model defined for the integrated prognostic model as follows: low, low‐intermediate, high‐intermediate, and high risk, demonstrating stronger prognostic separation across all end points (all *p *< 0.001) in our in‐house cohort and three validation cohorts. Collectively, the new feasible integrated prognostic stratification system contributes to accurate prognosis assessment in clinical routine and provides a new basis for the follow‐up treatment.

## Graphical abstract

1. Genetic subtypes based on molecular subtyping correlated with prognosis in DLBCL.

2. Integrated genetic subtype‐based IPI model revealed prognostic discrimination.

3. New four‐category risk model defined for integrated model showed diverse prognosis.

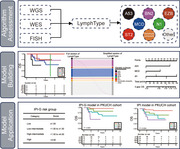

## 1 Introduction

Diffuse large B‐cell lymphoma (DLBCL), a heterogeneous disease, accounts for highest incidence in non‐Hodgkin lymphoma [1, 2]. Disease management is challenged by heterogeneity in clinical outcomes [3]. This malignancy exhibits significant clinical heterogeneity, characterized by various morphologic, genetic, and phenotypic features, which contribute to its variable prognosis and response to treatment. Combined immunochemotherapy and targeted therapy have changed the management of DLBCL over the past decade [4, 5, 6]. Despite significant progress in the treatment of DLBCL, a subset of patients still experiences poor prognosis.

In recent years, risk prognostic factors for DLBCL are increasingly being reported. International Prognostic Index (IPI) scoring system, including age, lactate dehydrogenase, performance status, stage, and extranodal involvement, is routinely used as global standard to predict prognostic stratification of DLBCL [7, 8]. While useful, IPI scoring system does not fully encompass the genetic heterogeneity observed in DLBCL. The integration of genomic or transcriptomic data into existing prognostic frameworks is essential for enhancing predictive accuracy and tailoring treatment approaches to individual patient profiles.

In order to better recognize the molecular mechanism of disease occurrence and development, genomic and transcriptomic abnormalities have recently been proved to be valuable prognostic biomarkers in multiple studies based on massively parallel next‐generation sequencing, playing a crucial role in the pathogenesis of DLBCL [9, 10, 11, 12, 13, 14]. Genomic studies have identified several recurrent genetic mutations in DLBCL, such as *MYD88* and *CD79B* [15]. These mutations have been associated with specific clinical and biological features of the disease. Additionally, gene expression profiling has led to the identification of distinct molecular subtypes of DLBCL, which have different responses to treatment [16]. A robust prediction model based on gene expression profiling facilitated the prognostic evaluation and risk stratification of patients with DLBCL [9]. Recent studies have also shown that defined genetic subtypes of DLBCL were both a potential target for drug efficacy evaluation, and an important biomarker for prognostic stratification [17, 18, 19, 20, 21, 22]. Although these subtyping methods have shed light onto the defined genetic subtypes, there remains a critical gap in the clinical application of genetic findings to improve patient stratification and treatment personalization. Up to now, an integrated prognostic model based on molecular typing algorithm and clinical features has not yet been well established.

Here, we aim to build a simplified algorithm to realize six defined genetic subtypes and propose a new integrated prognostic stratification system in newly diagnosed DLBCL, which could potentially lead to personalized treatment strategies and improved patient outcomes.

## 2 Results

### 2.1 Molecular Characteristics

**FIGURE 1:**
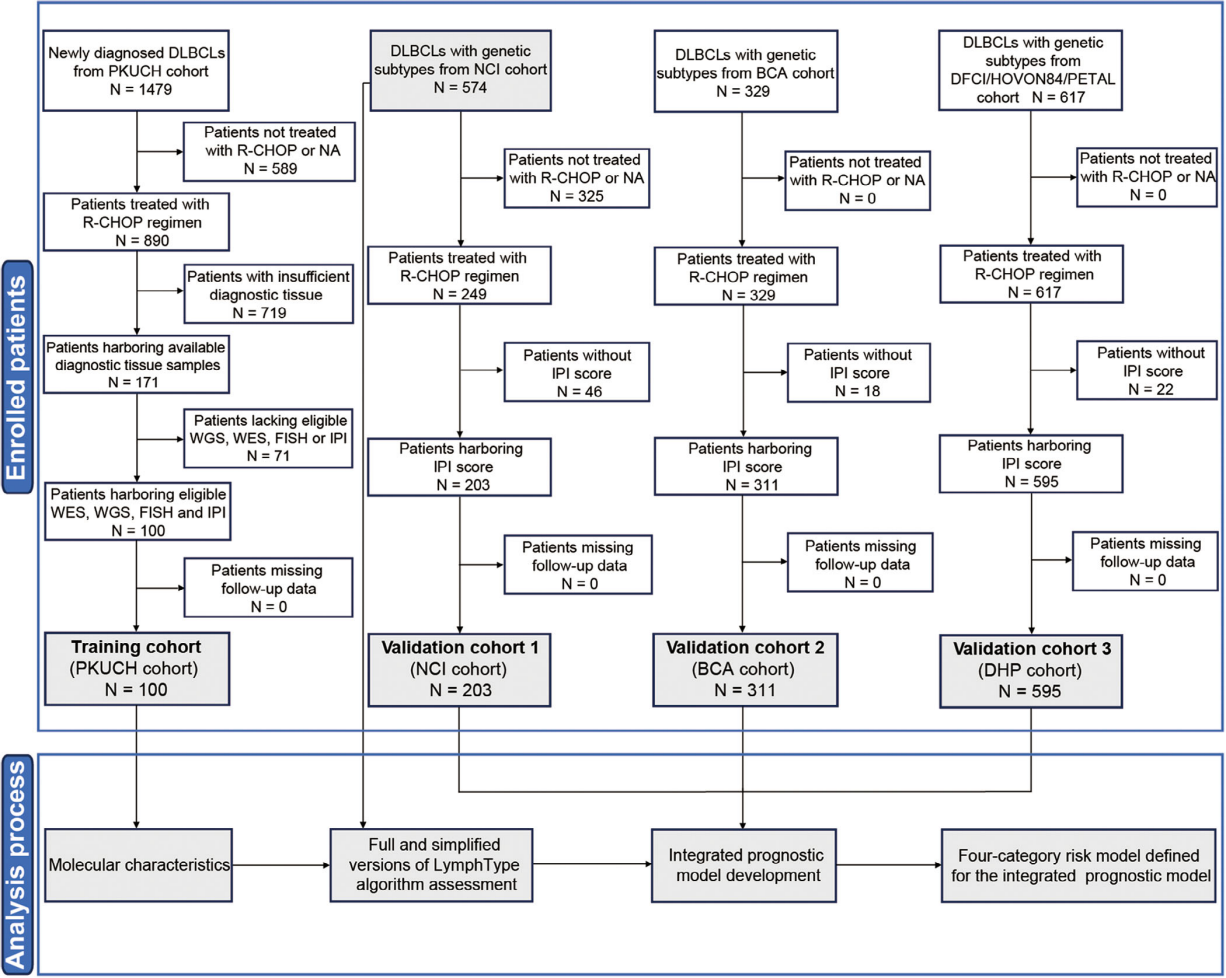
Enrolment of study cohort. DLBCL, diffuse large B‐cell lymphoma; R‐CHOP, rituximab, cyclophosphamide, doxorubicin hydrochloride, vincristine, and prednisone; WGS, whole genome sequencing; WES, whole exome sequencing; FISH, fluorescence in situ hybridization; IPI, International Prognostic Index; NA, not appliable.

The clinical characteristics of 100 newly diagnosed DLBCL patients in our Peking University Cancer Hospital & Institute (PKUCH) cohort are shown in Table S1, including 61 males and 39 females, with a median age of 57 years (range, 26–89). Forty‐one percent (41 out of 100) of the patients had internal lymph node lesions, and the rest 59.0% (59 out of 100) had external lymph node lesions, including primary testis, breast, and other sites. According to Hans cell of origin (COO) classification [23], 27.0% were germinal center B‐cell like (GCB). Flow chart of this study design was shown in Figure 1.

**FIGURE 2:**
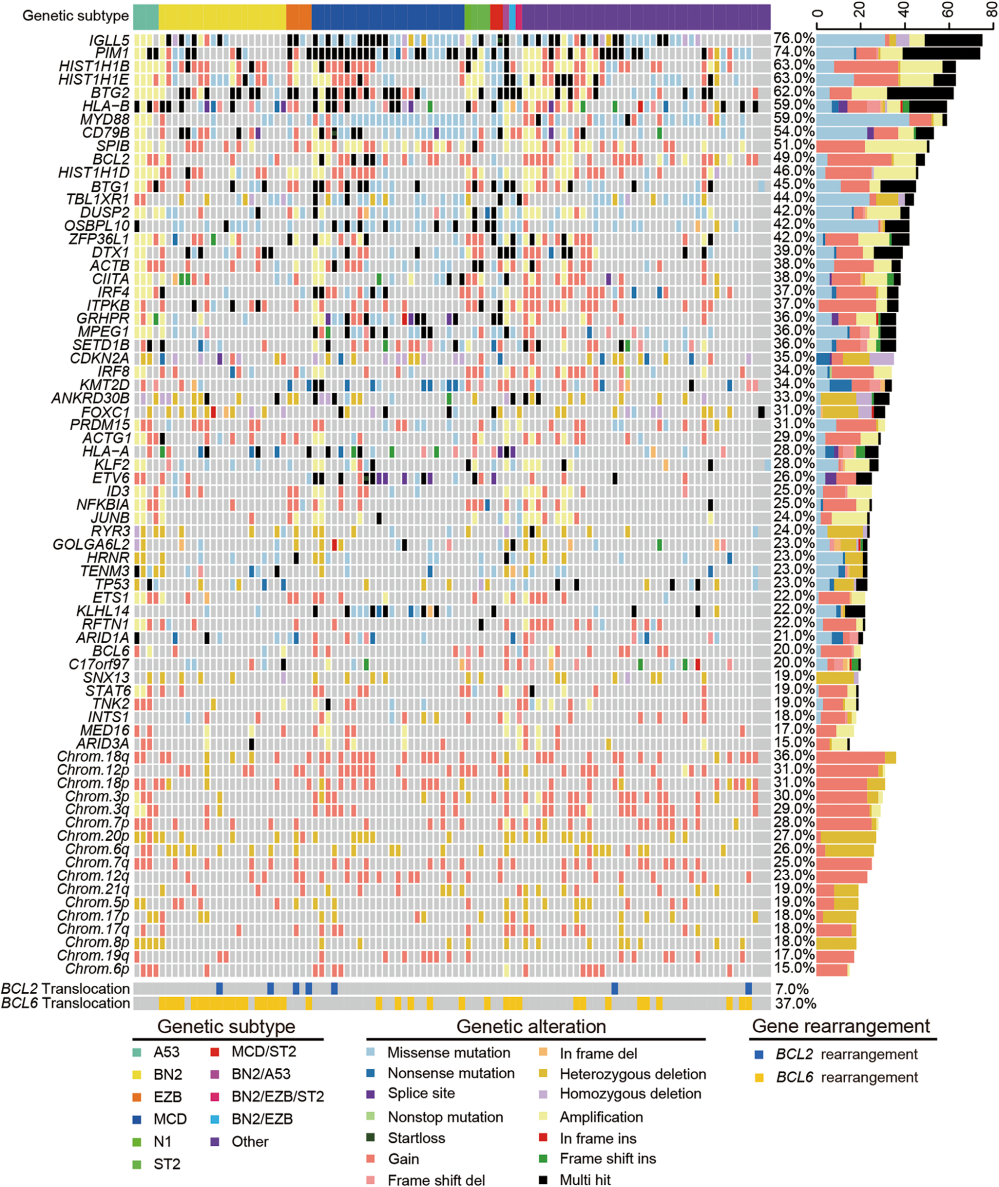
Genomic landscape in the training cohort. Heatmap shows top specific mutated genes in gene mutations, gene CNVs, and chromosomal CNVs in each patient, detected in ≥15% patients. CNV, copy number variation.

**FIGURE 3:**
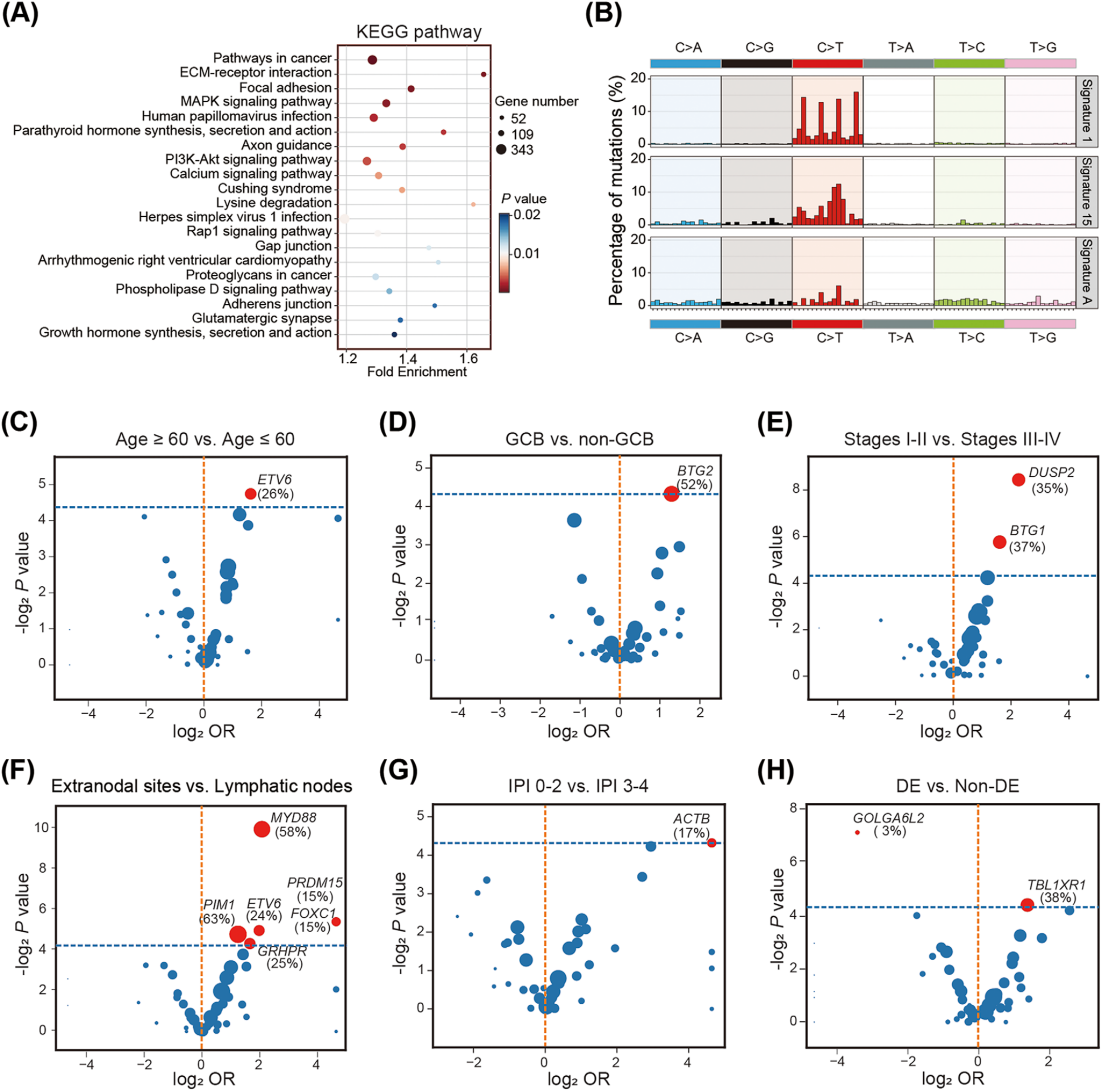
Genetic characteristics in the training cohort. (A) Bubble chart illustrates the mutation analysis of KEGG pathways. (B) Mutational signatures are displayed according to the 96 substitution classification defined by the different substitution class. Volcano plots display the correlation between gene mutations in mutated genes detected in ≥15% patients, and age (C), Hans COO classification (D), stage (E), invasion organ (F), IPI (G), and DE (H), respectively. KEGG, Kyoto Encyclopedia of Genes and Genomes; GCB, germinal center B‐cell like; IPI, International Prognostic Index; DE, BCL2/MYC double expressors.

Genomic landscape, including gene mutations, gene copy number variations (CNVs), and chromosomal CNVs, was established in Figure 2. Significant associations of gene mutations were discovered between mutated *MYD88* and mutations in *CD79B*, *PIM1*, *IGLL5*, *BCL2, ETV6*, *KLHL14*, *GRHPR*, and *TBL1XR1*, and between mutated *TP53* and *CD79B*, *PIM1*, and *IGLL5* mutations (all *p* < 0.05; Figure S1). Kyoto Encyclopedia of Genes and Genomes pathway enrichment analysis of the mutated genes revealed significant enrichment in pathways related to pathways in cancers, ECM–receptor interaction, focal adhesion, MAPK signaling pathway, and signal transduction and human papillomavirus infection (Figure 3A). Gene ontology enrichment results were shown in Figure S2, including biological process, cellular component, and molecular function analysis. Based on mutational signature analysis of 96 substitution patterns using the non‐negative matrix factorization algorithm [24], we discovered three mutational signatures, including Signature 1, Signature 15, and Signature A, related to age of cancer diagnosis, defective DNA mismatch repair, and unknown function, respectively (Figure 3B).

To explore the association of gene mutations with age, Hans COO classification, stage, invasion organ, IPI score, BCL2/MYC double expressors (DE), and treatment response, we performed gene‐related subgroup analyses. In different age groups, mutated *ETV6* was significantly present in elderly patients (*p* < 0.05; Figure 3C). Based on Hans COO classification, we discovered that mutated *BTG2* was significantly present in GCB group, compared with non‐GCB group (*p* < 0.05; Figure 3D). From the comparison of different stages, mutated *BTG1* and *DUSP2* were more common in patients with lower stages I–II (both *p* < 0.05; Figure 3E). Compared with primary lymphatic nodes, mutated *MYD88*, *ETV6*, *PIM1*, *PRDM15*, and *FOXC1* significantly existed in primary extranodal lymphomas (all *p* < 0.05; Figure 3F). Furthermore, in the IPI comparison groups, we concluded mutated *ACTB* was more familiar in lower IPI 0–2 group (*p* < 0.05; Figure 3G). Interestingly, mutated *TBL1XR1* was correlated with DE group, while *GOLGA6L2* mutation was related to non‐DE group (both *p* < 0.05; Figure 3H). In the analysis of treatment response, we combined complete response and partial response patients into response group, stable disease and progressive disease patients into nonresponse group for comparison, we found that no mutated genes were more distributed in response or nonresponse group (all *p* > 0.05).

### 2.2 Full and Simplified Versions of LymphType Algorithm Assessments

**FIGURE 4:**
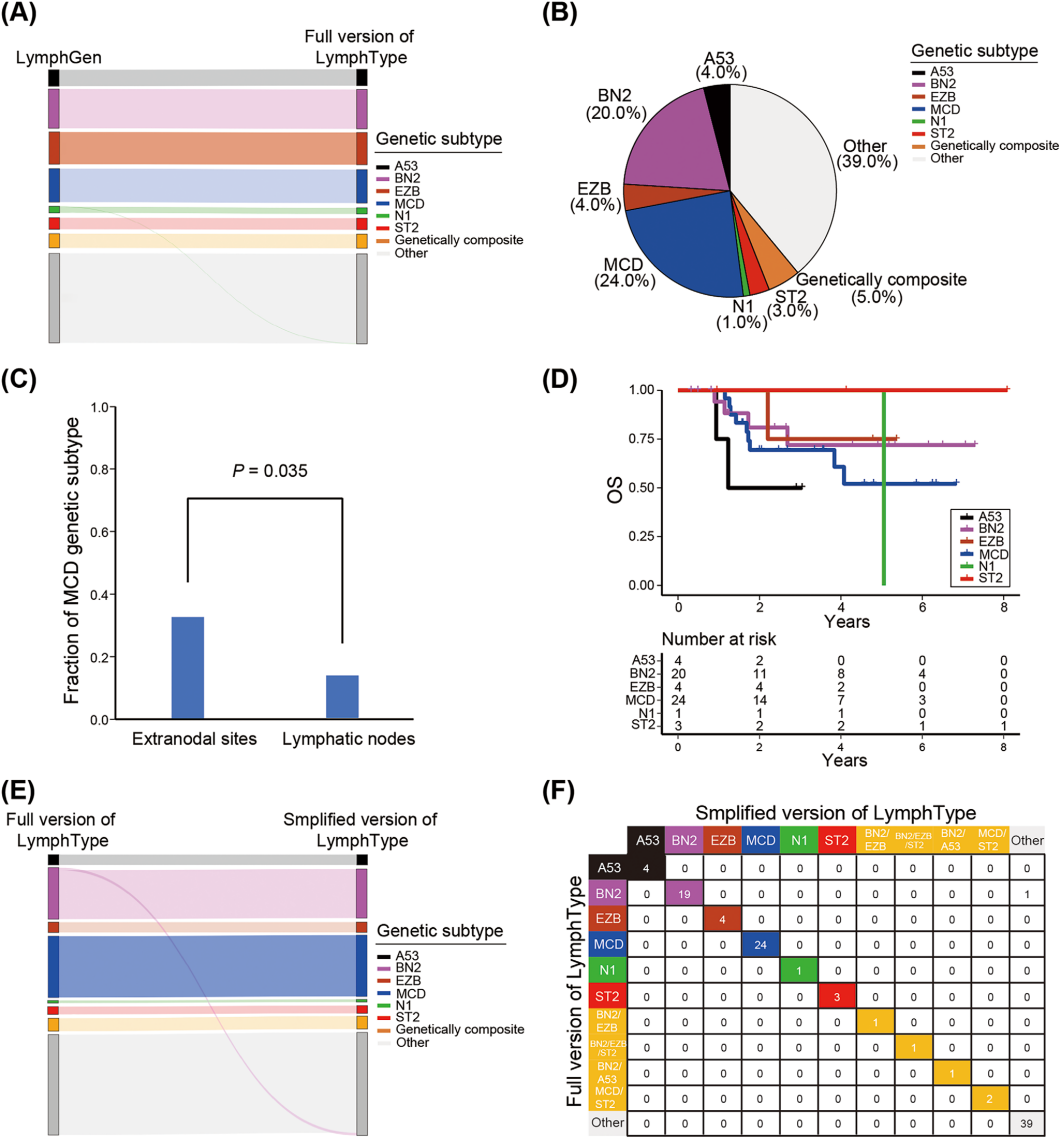
Performance evaluation of full and simplified versions of LymphType algorithm. (A) Sankey plot shows the comparison of the full version of LymphType and LymphGen algorithms in NCI cohort. (B) Pie chart displays the defined genetic subtypes based on full version of LymphType algorithm in PKUCH cohort. (C) Histogram shows the comparison between different genetic subtypes based on full version of LymphType algorithm in primary lymphatic node and extranodal lymphomas in PKUCH cohort. (D) Kaplan–Meier survival curve shows the prognostic effect of OS in PKUCH cohort according to full version of LymphType algorithm. (E) Sankey plot shows the comparison of the simplified version and full version of LymphType algorithm in PKUCH cohort. (F) Confusion matrix displays the number of matches in each defined genetic subtype based on the full and simplified versions of LymphType in PKUCH cohort. OS, overall survival.

According to the LymphGen algorithm on defined genetic subtypes of DLBCL [19], we implemented the optimization and construction of LymphType algorithm internally. From the comparison results of data classification in NCI cohort (*N* = 574) using LymphGen algorithm, we reached 99.8% consistency through inhouse algorithm. Among them, the classification consistency of A53, BN2, EZB, MCD, and ST2 subtypes reached 100%, genetic composite subtype reached 100%, and only one patient in the N1 classification on LymphGen algorithm was divided into “Other” subgroup (Figure 4A).

Next, we conducted molecular typing analysis of the LymphType algorithm on the results of 100 retrospective patients in our single‐center cohort. We discovered that the A53, BN2, EZB, MCD, N1, ST2, and genetically composite subgroups accounted for 4.0, 20.0, 4.0, 24.0, 1.0, 3.0, and 5.0% (Figure 4B). We further analyzed the genetic subtypes of DLBCL patients at different invasion organs. Compared with primary lymphatic nodes, the most common subtype was MCD in primary extranodal lymphomas (32.8 vs. 13.5%, *p* = 0.035; Figure 4C), which was consistent with previous study [19]. We then assessed the relationship between six defined genetic subtypes and prognosis in our training cohort. Overall, molecular typing tended to be an ideal way to distinguish overall survival (OS) in patients (Figures 4D and S3).

At present, the full version of our LymphType algorithm achieves accurate classification in DLBCL into six different defined genetic subtypes based on probabilistic method. However, due to the involvement of multiple omics analysis and intensive cost, the complex algorithm, including whole genome sequencing (WGS), whole exome sequencing (WES), and fluorescence in situ hybridization (FISH), brings some difficulties in clinical practice. Therefore, we propose a simplified algorithm for classification evaluation, achieved by WGS, targeted 74‐gene panel sequencing (Table S2), and FISH analysis. Compared with the full version of the LymphType algorithm, the accuracy of the simplified version is as high as 99.0%, as shown in Figure 4E,F. One patient (one out of 100) in the BN2 classification on full version of LymphType algorithm was classified into “Other” subgroup based on simplified LymphType algorithm, indicating the simplified algorithm has the nearly same prediction effect as the full version of the algorithm, which can be used in the subsequent research.

### 2.3 Integrated IPI and Simplified LymphType Algorithm Prognostic Model Development

We next evaluated optimal feature selection for prognostic model development of OS. Considering that composite subtype is composed of two or more single subtypes, we included specified subtype contained in composite subtype into each single subtype in prognostic analysis (Figure S3). In univariable Kaplan–Meier curve analysis to determine possible predictive factors associated with OS, we further analyzed age, performance status, stage, extranodal site, IPI scoring system, and genetic subtypes including MCD and A53 (both *p* < 0.2), as shown in Table S3.

**FIGURE 5:**
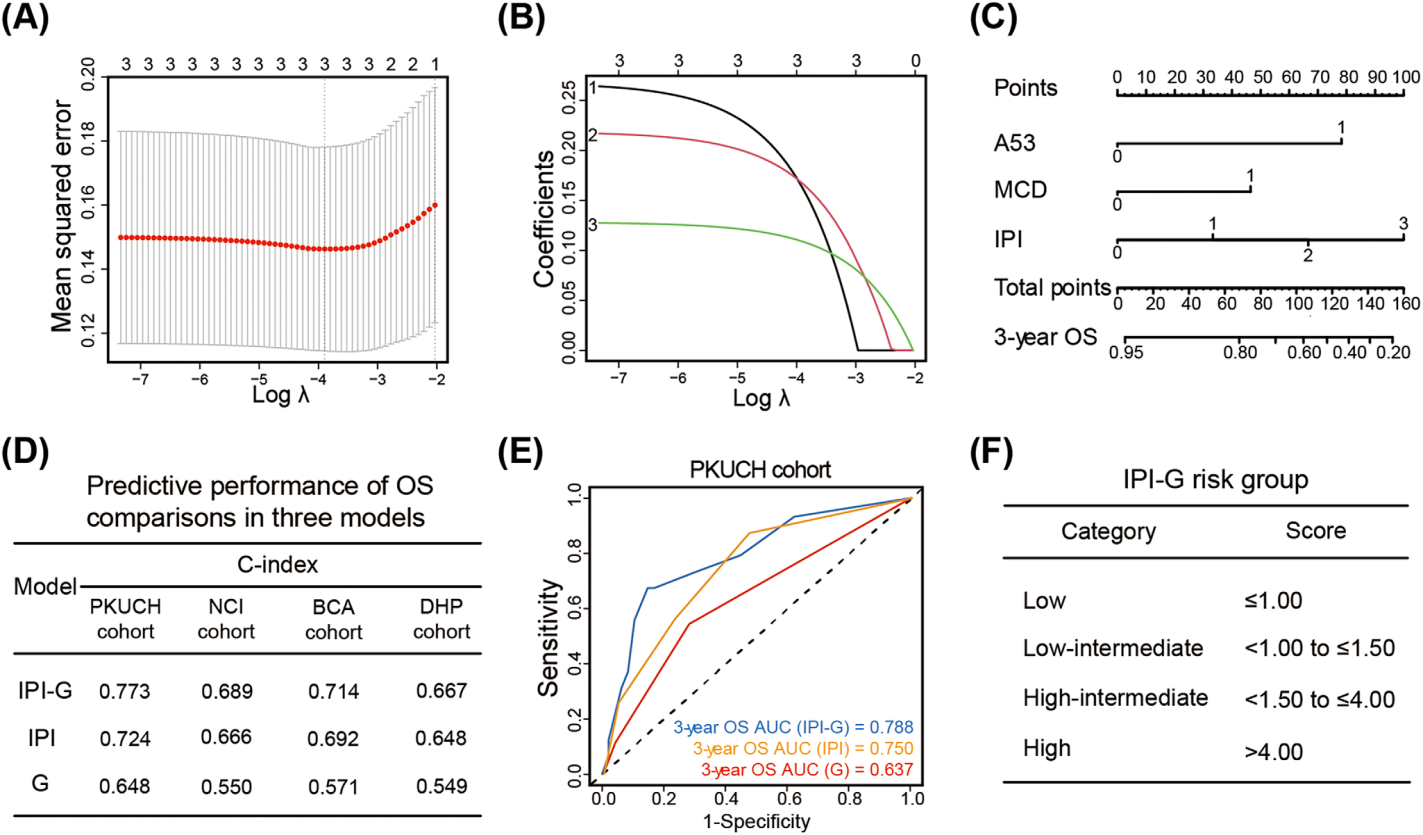
Predictive performance evaluation of the integrated IPI‐G prognostic model. (A) Ten‐fold cross validation curve for tuning parameter selection. The vertical and horizontal axis represents mean square error and λ, respectively. (B) The coefficient curve for tuning parameter. The vertical and horizontal axis represents the feature's coefficient and *λ*, respectively. (C) Nomogram model for predicting OS. (D) Predictive performance based on C‐index comparisons in three models, including IPI‐G, IPI, G models. (E) ROCs represent the predictive performances in the three models in the training PKUCH cohort. (F) Four‐category risk group defined for the integrated IPI‐G prognostic model. LASSO, least absolute shrinkage and selection operator; λ, lambda; OS, overall survival; C‐index, concordance index; IPI, International Prognostic Index; IPI‐G, genetic subtype‐based IPI; G, genetic subtype; ROC, receiver operating characteristic curve; LR, low risk; LIR, low‐intermediate risk; HIR, high‐intermediate risk; HR, high risk.

To prevent multicollinearity, we excluded variables discovered in univariable Kaplan–Meier curve analysis and also existed in IPI scoring system: age, performance status, stage, and extranodal involvement. Finally, three variables were incorporated in the integrated model based on IPI and two defined genetic subtypes, namely genetic subtype‐based IPI (IPI‐G) model, using least absolute shrinkage and selection operator (LASSO) Cox regression model, built as a weighted sum observed for each patient, based on coefficient profiles (Figure 5A,B). The integrated IPI‐G prognostic model was built including the weighted coefficients of these variables: IPI × 1.19 + MCD × 1.66 + A53 × 2.79 (IPI scored as 0–3, 0 denotes low risk [LR], 1 low‐intermediate risk [LIR], 2 high‐intermediate risk [HIR], and 3 high risk [HR]; two genetic subtypes mentioned above scored as 1). A prognostic nomogram that integrated all the three variables from the LASSO Cox regression model was constructed (Figure 5C). To discriminate and calibrate the nomogram for predicting OS, calibration curves were built to illustrate the optimal consistency for OS probability between predictions and actual observations in the training PKUCH cohort and validation NCI, BCA, and DHP cohorts (Tables S4–S6). All the calibration curves from the training cohort and three validation cohorts were well fitted (Figure S4).

To investigate the performance difference in predicting prognosis among IPI model, genetic subtype (G) model, and the new integrated IPI‐G model, we compared the discrimination ability with the concordance index (C‐index). The new IPI‐G prognostic model indicated best discrimination of OS with C‐index value of 0.773 in PKUCH cohort, compared with IPI score, classification algorithm model alone with C‐index value of 0.724 and 0.648, respectively. Similarity results were also seen in NCI, BCA, and DHP cohorts (Figure 5D). The area under curve (AUC) for predicting 3‐year OS displayed more excellent conformity based on the integrated IPI‐G model, compared with IPI model, and G model alone (AUC, 0.788 vs. 0.750 vs. 0.637) (Figure 5E). We further tried subgroup analysis in the PKUCH cohort, including DE subgroup and non‐GCB subgroup, and the performance of the IPI model was both demonstrated in the above two subgroups (Figure S5).

**FIGURE 6:**
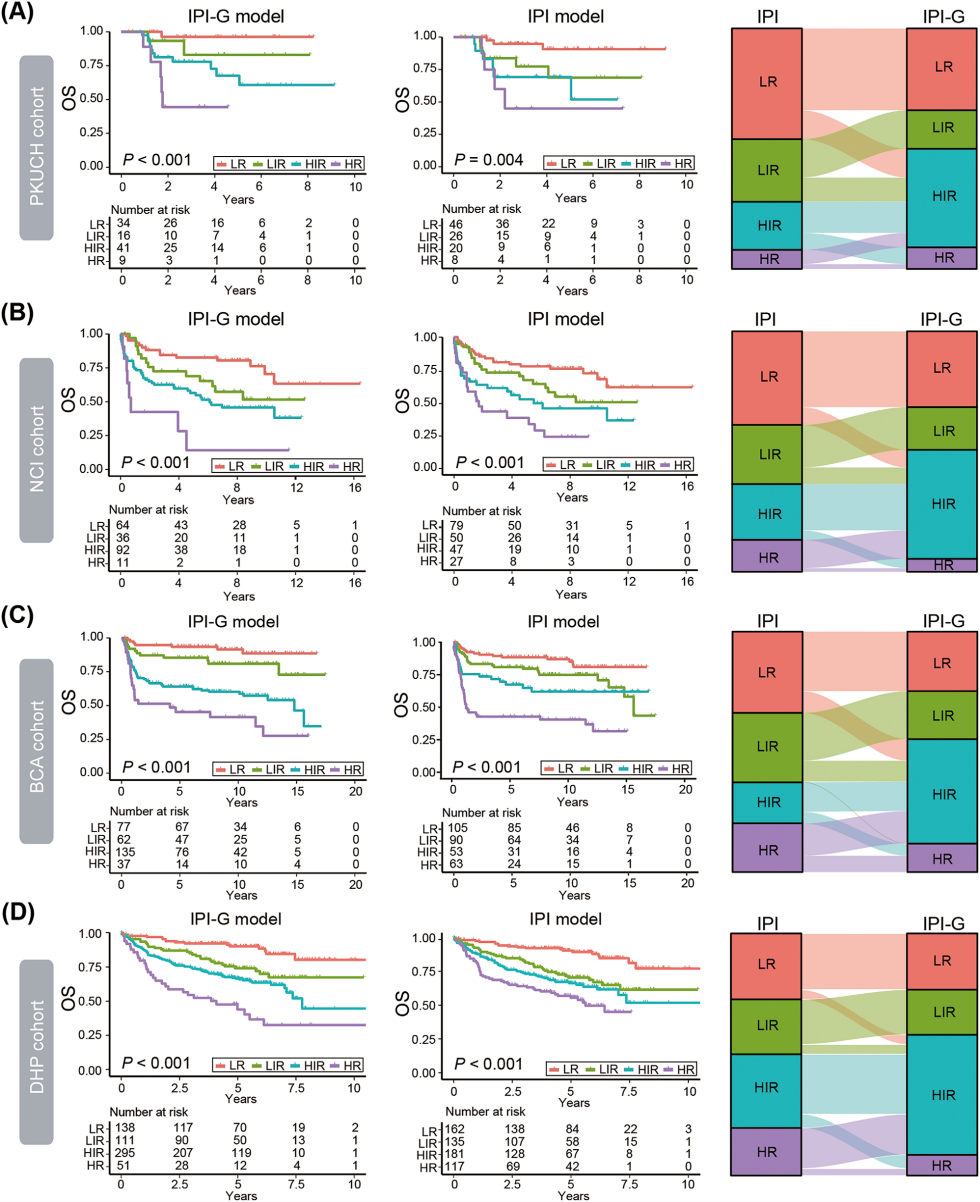
The four‐category risk model defined for the integrated IPI‐G prognostic model. Kaplan–Meier survival curves and Sankey plots based on four‐category integrated IPI‐G prognostic model, compared with IPI model, in the training PKUCH cohort (A), and the validation NCI cohort (B), BCA cohort (C), and DHP cohort (D), respectively. OS, overall survival; IPI, International Prognostic Index; IPI‐G, genetic subtype‐based IPI; LR, low risk; LIR, low‐intermediate risk; HIR, high‐intermediate risk; HR, high risk.

As the four‐category IPI scoring system has guided clinical studies, we subsequently established a four‐category risk model defined for the integrated IPI‐G prognostic model mainly based on the maximally selected log‐rank statistics as follows: LR, LIR, HIR, and HR, scored at ≤1.00, <1.00 to ≤1.50, <1.50 to ≤4.00, and >4.00, respectively (Figure 5F). Our new four‐category risk IPI‐G model demonstrated stronger prognostic separation across all end points and especially to solve the cross problem of partial survival curves, compared with the four‐category IPI model, in the training PKUCH cohort from our center (Figure 6A) and the validation NCI, BCA, and DHP cohorts (Figure 6B–D). Due to the limited sample size of each cohort, we combined the four cohorts for data analysis (*N* = 1209). We discovered the crossover phenomenon was existed between LIR and HIR survival curves in the IPI model, but the IPI‐G model can successfully enhance patient stratification (Figure S6).

## 3 Discussion

In the present study, we built a simplified algorithm to realize six defined genetic subtypes based on WGS, targeted 74‐gene panel sequencing, and *BCL2* or *BCL6* rearrangement status, and first developed a new integrated prognostic stratification system, combined IPI scoring system and simplified defined genetic subtypes in DLBCL.

Our research confirmed the landscape of genetic alterations, including gene mutations, gene CNVs, chromosomal CNVs, and *BCL2* or *BCL6* rearrangements, in newly diagnosed DLBCLs. The most frequently mutated genes discovered in our cohort were *IGLL5* (76.0%), *PIM1* (74.0%), *HIST1H1B* (63.0%), *HIST1H1E* (63.0%), and *BTG2* (61.1%), consistent with the results of previous Sánchez‑Beato et al.’s study [25]. The prognostic value of gene mutations has been well reported in several studies. DLBCL patients with *TP53* mutations harbored shorter survival [12, 26–28]. Mutations in *CD79B*, *ETS1*, and *CD58* had a significantly inferior survival [25]. *NOTCH1* mutations, independent of established clinical variables, were significantly associated with poorer survival [29]. A related study have shown that patients can be stratified via the gene expression profiling‐based model [9]. In our study, we identified several genes that align with those reported in the referenced article, such as *HLA*‐*B*, *ZFP36L1*, and *ITPKB*. While the key genes in our mutational model, such as *TP53*, *MYD88*, and *CD79B*, were not discovered in the expression gene model. However, whether the above gene mutations are related to gene expression needs to be further studied in basic research.

According to gene expression profiling, the well‐known COO classification divided DLBCLs into activated B cell and GCB subtypes, closely related to the prognosis [30, 31]. The emergence of the Hans classification, with immunohistochemical analysis of CD10, BCL6, and MUM1, made COO classification an easier method in clinical practice [23]. Molecular subtyping studies in DLBCL based on genetic information have been reported gradually in recent years [12, 17, 19, 20, 25, 32], leading to the proposals of novel defined genetic subtypes determined by distinct genetic patterns. In 2018, Staudt et al. [17] first identified four prominent genetic subtypes, including MCD, BN2, N1, and EZB, in 574 DLBCL patients, providing a potential classification for precision‐medicine strategies. Almost at the same time, Shipp et al. [18] performed a comprehensive genetic analysis in 304 primary DLBCLs and discovered five distinct DLBCL subsets, including Cluster 0–5. In 2020, the above research group, Staudt et al. [17], then proposed a seven‐classification algorithm, named LymphGen algorithm, containing A53, BN2, EZB‐MYC^+^, EZB‐MYC^−^, MCD, N1, and ST2. They discovered distinct genetic subtypes harbored different prognosis and pathway dependencies, suggesting that drug use could be guided according to different genetic subtypes [19]. The Phoenix trial concluded that MCD or N1 subtypes of DLBCL patients (aged ≤60 years) experienced more significantly improved event‐free survival treated with ibrutinib plus rituximab, cyclophosphamide, doxorubicin hydrochloride, vincristine, and prednisone (R‐CHOP) regimen, compared with R‐CHOP alone [33].

Based on LymphGen algorithm [19], several research groups have done optimization and research on this basis, and proposed simplified versions of the algorithm, including Sakaida et al. [32] from a Japanese cohort, Sánchez‐Beato et al. [25] from a Spain cohort, and Zhao et al. [20] from a Chinese cohort. Different from other studies mentioned above, the samples of enrolled patients in our study were all adopted under unified data acquisition conditions, such as the same sequencing panel for WES and sequencing platform for WES and WGS, to ensure the comparability of results. The integrated IPI‐G model based on our study is more convincing. Our six defined genetic subtypes, including A53, BN2, EZB, MCD, N1, and ST2, were similar to those previously reported, and the most frequent subtype in our PKUCH cohort was MCD, characterized by cooccurrence of *MYD88* and *CD79B* mutations [13, 17, 19]. Unfortunately, since our previous design did not incorporate the *MYC* rearrangement status, our algorithm could not further classify the EZB subtype into EZB‐MYC^+^ or EZB‐MYC^−^. The complexity of algorithms mentioned above, containing the large number of gene mutations, gene CNVs, chromosomal CNVs, and rearrangements used to define the genetic subtypes, made it challenging to perform them in the real‐world clinical routine. Therefore, the proposed simplified version of the LymphType algorithm, with 99.0% consistency of full version algorithm, which was close agreement with that of LymphGen (99.8%), can be more convenient for clinical use, as the simplified version of LymphType changes the WES involved in molecular typing to multigene panel sequencing. Since the core determinant of A53 subtype is chromosomal CNV [19], WGS sequencing data can be used to identify chromosomal CNV more accurately. The exact classification of A53 is also conducive to the accurate determination of other subtypes. Therefore, our simplified version of the algorithm incorporates WGS sequencing. From the point of view of clinical translation, our simplified version of the algorithm overcame magnificent obstacles, including complicated computational expertise and intensive cost. In terms of the sequencing process, it is relatively simple to complete the construction of the experimental sequencing libraries with the same tissue samples, which are used for both WGS and WES sequencing, respectively.

The survival rates of DLBCL patients with different defined genetic subtypes were revealed to be diverse, and both MCD and A53 subtypes were observed to be a poorer prognostic subtype in our cohort, consistent with previously studies [12, 17–19, 22, 34]. Highlighting the significance of our finding, although the genetic mutation analysis in the DLBCL prognosis has been reported, the prognosis assessment model of gene mutations combined with clinical characteristics, such as IPI, has not been well explored. The new IPI‐G nomogram model exhibited excellent prediction ability with a C‐index of 0.773 better than IPI score system or classification algorithm alone, indicating IPI score combined with molecular subtyping plays an important role in prognostic stratification. Considering the feasibility in clinical practice, we classified the integrated model into four categories, and found the four‐category model could effectively distinguish the prognosis of DLBCL patients, especially for patients with LIR and HIR based on IPI model. Genetic subtyping results help stratify patient prognosis and select targeted drugs, ultimately enhancing clinical benefits. For example, MCD‐subtype patients can be treated with Bruton's tyrosine kinase inhibitors to enhance efficacy and prognosis [21, 33]. In the future, newly diagnosed patients should undergo molecular typing tests for drug selection and comprehensive prognostic evaluation. Our study focused solely on an in‐depth analysis of DNA data. Given the prognostic value of RNA expression results [9], we conclude that combining DNA and RNA data may more effectively differentiate patient prognosis. However, this requires further verification.

There are several limitations in our current study. First, this was a single‐center retrospective study. Second, the integrated model was developed in a relatively small cohort despite external validations. Third, the role of genetic subtyping in drug efficacy was not investigated in this study. A multicenter prospective study is needed to verify the feasibility of this model and drug efficacy evaluation in the future.

In summary, we build a new feasible integrated prognostic stratification system, consisting of IPI scoring system and simplified defined genetic subtypes, in newly diagnosed DLBCL, contributing to accurate prognosis assessment in clinical routine and providing a new basis for the follow‐up treatment.

## 4 Methods and Materials

### 4.1 Study Cohort

A total of 100 newly diagnosed DLBCL patients with eligible WGS, WES, and FISH testing data per World Health Organization criteria were enrolled in this retrospective study at PKUCH from January 2014 to January 2023. Diagnostic confirmation was independently performed by two expert hematopathologists. All patients had no bone marrow infiltration at diagnosis, uniformly treated with R‐CHOP like regimen, and had long‐term follow‐up at March 2024. Rearrangements of *BCL2* and *BCL6* were assessed by FISH analysis based on formalin‐fixed paraffin embedded (FFPE) tissues. The study was approved by the Ethics Committee at PKUCH in accordance with the Declaration of Helsinki. Informed consents were obtained from patients.

### 4.2 Sample Collection, Processing, and Sequencing Procedure

Fifty‐eight percent (58 out of 100) of patients had FFPE tissues with a paired normal specimen, and the remaining 42.0% (42 out of 100) of patients owned only FFPE tissues. Peripheral blood samples were selected as a source for germline DNA identification. Sample collection, processing, and sequencing procedure details were shown in Supporting Information: Materials and Methods.

For mutation calling from WES data, MuTect2 [35] were performed for small insertions and deletions, and mutations were annotated with ANNOVAR software [36]. For copy number analysis from WES data, we conducted in house algorithm for gene CNVs. In brief, whole exomes were divided into adjacent and nonoverlapping bins based on the exons of each gene, and the coverage of each bin was calculated. The coverage bias related with GC content of the reference genome was normalized. Then, we build a baseline based on 50 healthy individuals and calculated the residuals of each bin over the baseline using a LOESS‐based method. For structural variants from R‐CHOP data, arm‐level CNVs were identified by WisecondorX [37]. The variants were further filtered by recurrent sequencing artifacts and germline events in an in‐house list based on approximately 1000 tissue and peripheral blood samples as normal pool from nonlymphoma patients with the same WES sequencing panel. We also developed an algorithm called SomaticFinder to analyze somatic mutations based on tumor‐only samples (Supporting Information: Materials and Methods and Figure S5).

### 4.3 LymphType Algorithm Development

The goal of our algorithm, named LymphType, is to achieve six defined genetic subtypes using WES, WGS, and FISH data, based on LymphGen [19]. The core of the LymphType algorithm is to realize molecular typing by gene mutations, gene CNVs, chromosomal CNVs, and *BCL2* or *BCL6* rearrangements. Gene mutations were obtained from WES, including missense, nonsense, silent, and frameshift mutations. Gene CNVs were derived from WES. Chromosomal CNVs were gained from WGS, including amplification, gain, heterozygous deletion, and homozygous deletion. LymphType algorithm divided the patients into six single genetic subtypes, including A53, BN2, EZB, MCD, N1, and ST2, and several genetically composite subtypes.

### 4.4 External Validation Cohorts

Three external validation cohorts were enrolled in this study (Figure 1). Validation cohort 1 (NCI cohort) was used for LymphType algorithm development (*N* = 574), and the integrated IPI‐G prognostic model and the four‐category risk model defined for the integrated IPI‐G prognostic model validations (*N* = 203) [19]. Validation cohort 2 (BCA cohort) and validation cohort 3 (DFCI/HOVON84/PETAL (DHP) cohort) were both performed to validate the integrated IPI‐G prognostic and the four‐category risk models mentioned above (*N* = 311, *N* = 595, respectively) [22, 38].

### 4.5 Statistical Analysis

Statistical tests were performed using SPSS (version 22.0) or R package (version 4.3.1). Continuous variables were compared using Mann–Whitney or Wilcoxon test. Categorical variables were compared using chi‐square or Fisher's exact test. OS was measured from the date of diagnosis to death or last follow up. Survival analyses were evaluated with the Kaplan–Meier curves using the log‐rank test. Variables with a *p *< 0.2 in univariable Kaplan–Meier curve analysis were selected for LASSO Cox regression analysis for data dimensionality reduction and variable selection, improving prediction accuracy and interpretation. All *p* values, two‐sided, less than 0.05 were considered statistically significant.

## Author Contributions

W. L., Y. S., S. C., F. L., and J. Q. designed the study and approved the final manuscript. L. M., J. D., C. Z., and J. Z. collected the clinical sample and data. S. Y., L. C., H. Wu, H. Wang, and H. C. performed the sequencing platform. J. D., C. Z., and L. L. analyzed the data. L. M., J. D., L. L., and J. Q. interpreted the results. L. M., J. D., F. L., and J. Q. drafted and revised the manuscript. All authors have read and approved the final manuscript.

## Ethics Statement

The study was approved by the Ethics Committee at Peking University Cancer Hospital & Institute in accordance with the Declaration of Helsinki (approval number: 2022KT163). Informed consents were obtained from patients.

## Conflicts of Interest

Authors Jiayue Qin, Lixia Liu, Shunli Yang, Libin Chen, Hong Chen, Feng Lou, and Shanbo Cao are the employees of Acornmed Biotechnology Co., Ltd., but has no potential relevant financial or nonfinancial interests to disclose. The other authors declare no conflicts of interest.

## Supporting information



Supporting Information

## Data Availability

The datasets generated and/or analyzed during the current study are available in Genome Sequence Archive under project PRJCA035451.
