# Supplementary material for: Genetic Subtype‐Based International Prognostic Index Prognostic Model in Diffuse Large B‐Cell Lymphoma
Source: MedComm (2020). 2025 Jun 16;6(7):e70190. doi: 10.1002/mco2.70190 (PMC12171059; doi:10.1002/mco2.70190)
Supplement: Supplementary file 1 — Supporting Information [file MCO2-6-e70190-s001.docx]

Supplementary information for

Genetic subtype-based International Prognostic Index prognostic model in diffuse large B-cell lymphoma

**This file includes:**

Supplementary methods and materials

Figures S1 to S7

Tables S1 to S6

**Supplementary methods and materials.** Sample collection, processing and sequencing procedure. SomaticFinder algorithm development.

**Figure S1.** Gene mutation association analysis with mutated genes detected in ≥ 15% patients.

**Figure S2.** Go enrichment of mutated genes, including biological process (A), cellular component (B), and molecular function (C).

**Figure S3.** Kaplan-Meier survival curves based on simplified LymphType algorithm in the training PKUCH cohort.

**Figure S4.** Calibration plots in the training PKUCH cohort from our center (A) and the validation cohorts from NCI dataset (B), BCA dataset (C) and DHP cohort (D).

**Figure S5.** Predictive performance of OS comparisons in three models in two subgroups in the training PKUCH cohort, including DE subgroup and non-GCB subgroup.

**Figure S6.** Kaplan-Meier survival curves and Sankey plot based on four-category integrated IPI-G prognostic model, compared with IPI model, in the combined 4 cohorts, including PKUCH cohort, NCI cohort, BCA cohort, and DHP cohort.

**Figure S7.** ROC of SomaticFinder in the training set (A) and testing set (B).

**Table S1.** Patient baseline clinical characteristics in the training PKUCH cohort.

**Table S2.** Targeted 74-gene panel for simplified LymphType algorithm.

**Table S3.** Univariate Kaplan-Meier curve analysis of OS in the training PKUCH cohort.

**Table S4.** Comparisons of clinical features between the training cohort and validation cohort 1.

**Table S5.** Comparisons of clinical features between the training cohort and validation cohort 2.

**Table S6.** Comparisons of clinical features between the training cohort and validation cohort 3.

**Supplementary methods and materials**

**Sample collection, processing and sequencing procedure**

NGS tests were conducted in the centralized lab (Acornmed Biotechnology Co., Ltd., China). Genomic DNA (gDNA) was extracted from FFPE tissue and peripheral blood sample according to the instructions of UPure FFPE Tissue DNA Kit (Biokeystone, China) and MagCore Genomic DNA Whole Blood Kit (Concert, China), respectively. The concentration of gDNA was measured using Qubit 4.0 Fluorometer (Thermo Fisher Scientific, USA) and the gDNA was quantified by Real-time PCR (Thermo Fisher Scientific, USA).

Next, sequencing libraries were prepared from fragmented gDNA (300 ng) using the KAPA DNA Hyper Prep Kit (Roche, Switzerland). The DNA libraries were captured with the KAPA HyperExcome (Roche, Switzerland) for WES with an average depth of 545x (inter quartile range 396-647). DNA libraries for WES and WGS were both sequenced in DNBSEQ-T7 instruments (BGI Genomics, China). The filtering raw variant rules for WES and WGS are as follows: mapping quality ≥ 30 and base quality ≥ 30 for both, average sequencing depth on target per sample ≥ 200x and ≥ 1x, respectively. Burrows-Wheeler alignment (BWA, version 0.7.12) was performed to align the trimmed reads the Human Genome Reference Consortium build 37 (GRCh37).^1^ MarkDuplicates tool from Picard was used to mark the PCR duplicates. IndelRealigner and BaseRecalibrator from Genome Analysis Toolkit (GATK, version 3.8) were applied for realignment and recalibration of the BWA data, respectively.^2^

**SomaticFinder algorithm development**

Due to lack of sufficient paired control samples, we developed an algorithm, called SomaticFinder, to identify somatic mutations base on tumor-only samples. Samples with paired controls were analyzed for mutations according to the paired sample, and samples without paired controls were analyzed for mutations using SomaticFinder.

In brief, we divided the 67 paired samples from PKUCH cohort into a training set and a testing set in a ratio of 7:3. Those calls that passed statistical filters and fell into coding regions were used to train and test the classifier. Variants were filtered based on the following criteria: (1) minimum coverage of at least 50×; (2) minimum alt reads of at least 4×; (3) variant allele frequency (VAF) < 0.02; (4) in-house background constructed from 1000 cases of peripheral blood and blacklist filter of untrusted mutations; (5) reducing sequencing-related artifacts based on iDES method.^3^

For germline mutations detected in both tumor and control samples, we only keep the mutations obtained after filtering above. For somatic mutations only detected in tumor samples, further filtering was performed based on the database, including variants collected in dbSNP (version 142), variants with population frequency < 0.0001 in the ExAC database. For the training set, we obtained 11026 somatic mutations and 22110 germline mutations from 48 pairs of samples. For the test set, we harbored 3558 somatic mutations and 91804 germline mutations from 19 pairs of samples.

For each mutation, the features were extracted and used for model training. Some of the features came from external databases, including (1) the presence of a candidate variant in the dbSNP; (2) the presence of a candidate variant in the COSMIC (v88); (3) the presence of a candidate variant in the ExAC; (4) toxicity scores provided by multiple models (SIFT, Polyphen2_HDIV, LRT, MutationTaster, MutationAssessor, FATHMM, RadialSVM, LR_score, VEST3_score, CADD, GERP++_RS, phyloP46way_placental, phyloP100way_vertebrate, SiPhy_29way_logOdds), considering evolutionary factors such as the degree of conservation of the affected residue;^4^ and (5) sequence context was defined as the three-base sequence comprising. The other features were the variants themselves, including (1) the fraction of samples carrying that particular variant over the total number of samples in the peripheral blood background. Variants with high sample frequencies are more likely to be germline polymorphisms; (2) VAF; (3) the VAF significantly related to the Flanking regions; (4) two base sequence that contains the reference (wild type) and the newly introduced variant base of the mutation. A total of 38 mutation-associated features were used for model construction. Due to the fact that most toxicity scores are composed of non-synonymous mutations, we only choose non-synonymous mutations for model construction and prediction. Missing value processing using Multivariate Imputation by Chained Equations (MICE),^5^ which is a multiple imputation method where missing values are filled in order to create a complete dataset.

An in-house Python script based logistic regression (LR) model was used for the development of machine learning models.^6, 7^ To mitigate overfitting, 10 bootstraps of the training cohort were performed for hyperparameter optimization wherein each bootstrap randomly selected 70% of the training cohort for model training and the remaining 30% for model validation. The average probability of the resulting 10 sub-models was used as the final prediction score of a sample. Variants were described by ten features that ultimately contributed to subsequent machine learning training and test steps.

The receiver operating curve (ROC) for the training set and testing set was depicted in Figure S7. The AUC in the training set was 0.981 (95% CI: 0.980-0.981), and 0.990 (95% CI: 0.989-0.991) in the testing set, respectively.

**References**

1. Li H, Durbin R. Fast and accurate short read alignment with Burrows-Wheeler transform. *Bioinformatics*. 2009;25(14):1754-60.

2. McKenna A, Hanna M, Banks E, et al. The Genome Analysis Toolkit: a MapReduce framework for analyzing next-generation DNA sequencing data. *Genome Res*. 2010;20(9):1297-303.

3. Newman AM, Lovejoy AF, Klass DM, et al. Integrated digital error suppression for improved detection of circulating tumor DNA. *Nat Biotechnol*. 2016;34(5):547-555.

4. Flanagan SE, Patch AM, Ellard S. Using SIFT and PolyPhen to predict loss-of-function and gain-of-function mutations. *Genet Test Mol Biomarkers*. 2010;14(4):533-7.

5. Azur MJ, Stuart EA, Frangakis C, Leaf PJ. Multiple imputation by chained equations: what is it and how does it work? *Int J Methods Psychiatr Res*. 2011;20(1):40-9.

6. Dou B, Zhu Z, Merkurjev E, et al. Machine Learning Methods for Small Data Challenges in Molecular Science. *Chem Rev*. 2023;123(13):8736-8780.

7. Greener JG, Kandathil SM, Moffat L, Jones DT. A guide to machine learning for biologists. *Nat Rev Mol Cell Biol*. 2022;23(1):40-55.


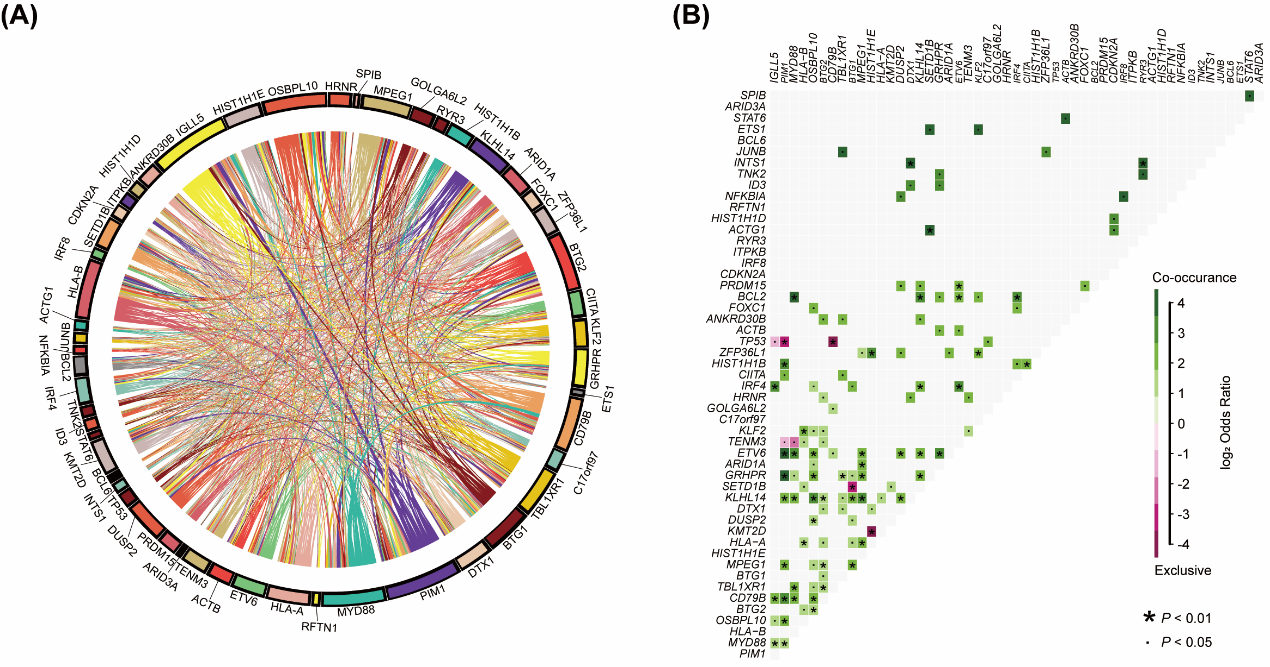


**Figure S1.** Gene mutation association analysis with mutated genes detected in ≥ 15% patients. (A) Circos diagram displays gene association, according to the relative frequency and pairwise co-occurrence of mutated genes. (B) Diagram shows pairwise gene mutation correlations with the odds ratio of the correlation coded by different colors.

**
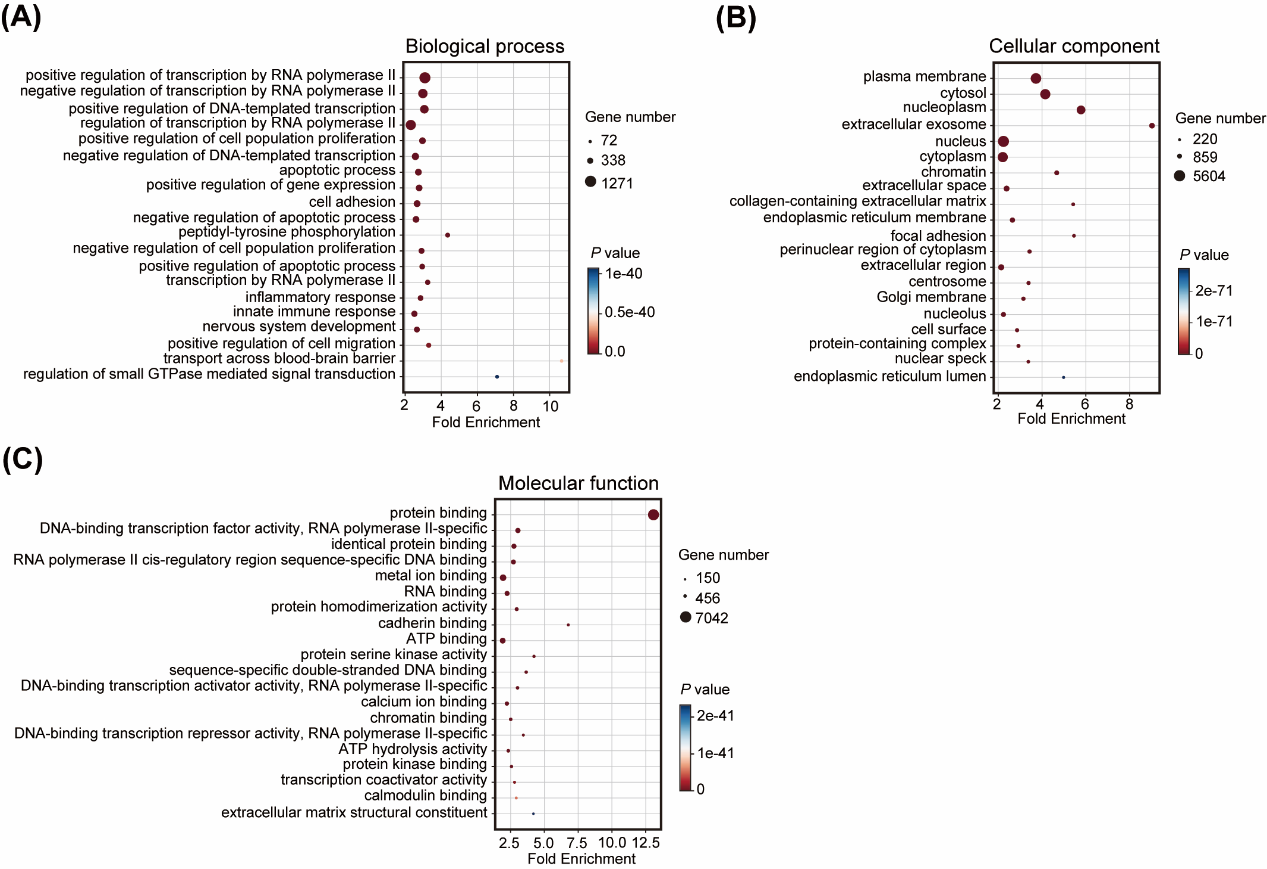
**

**Figure S2.** Go enrichment of mutated genes, including biological process (A), cellular component (B), and molecular function (C). GO, Gene ontology.

**
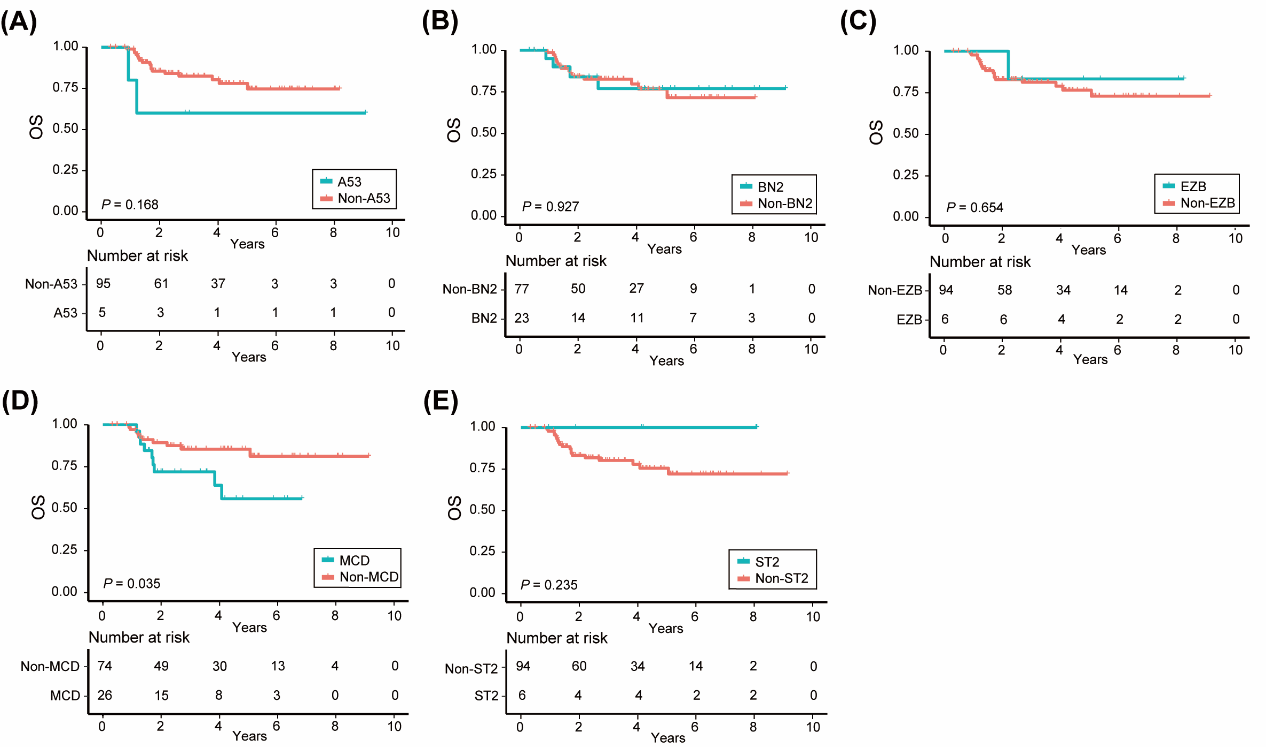
**

**Figure S3.** Kaplan-Meier survival curves based on simplified LymphType algorithm in the training PKUCH cohort**.** Specified subtype contained in composite subtype was considered into each single subtype. OS, overall survival.

**
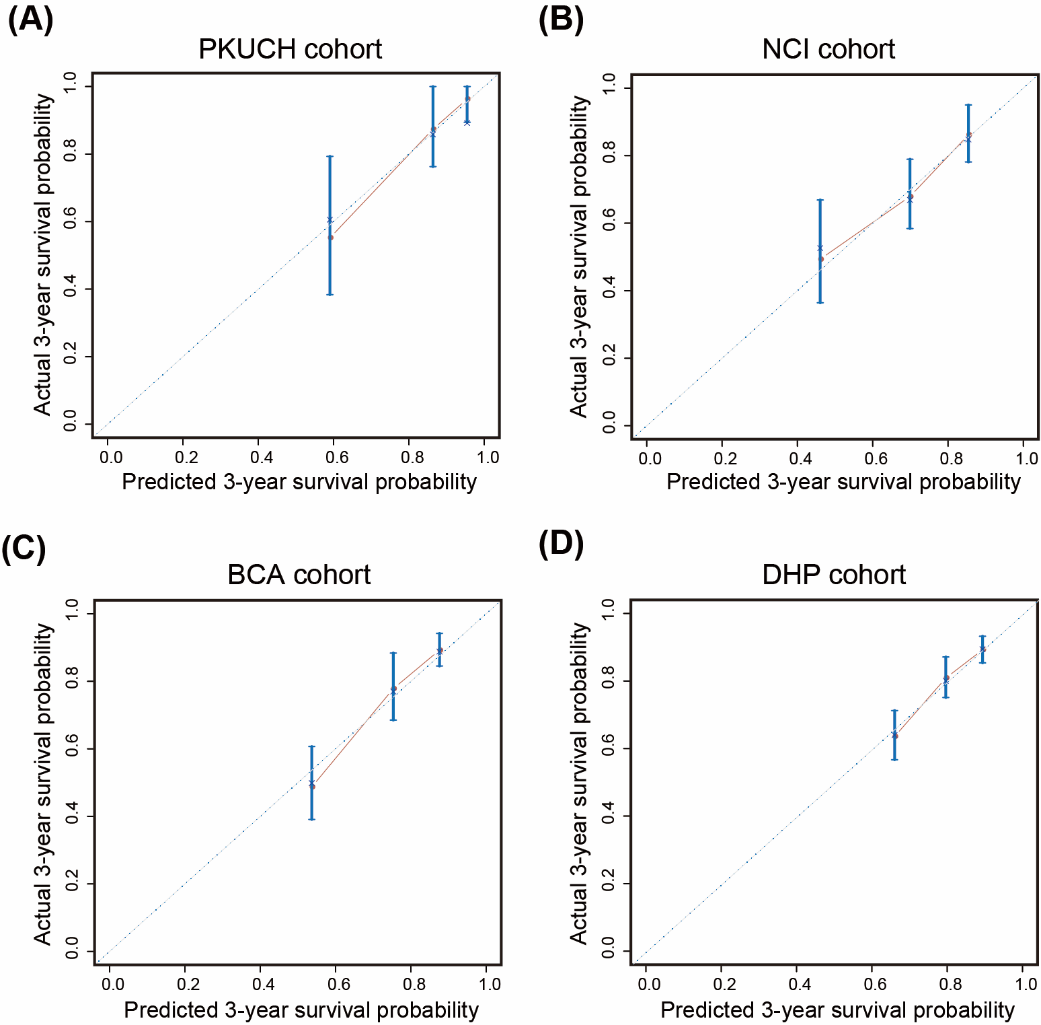
**

**Figure S4.** Calibration plots in the training PKUCH cohort from our center (A) and the validation cohorts from NCI dataset (B), BCA dataset (C) and DHP dataset (D). Ideal, observed, and optimism corrected values were marked with gray solid line, red solid line, blue dotted line.

**
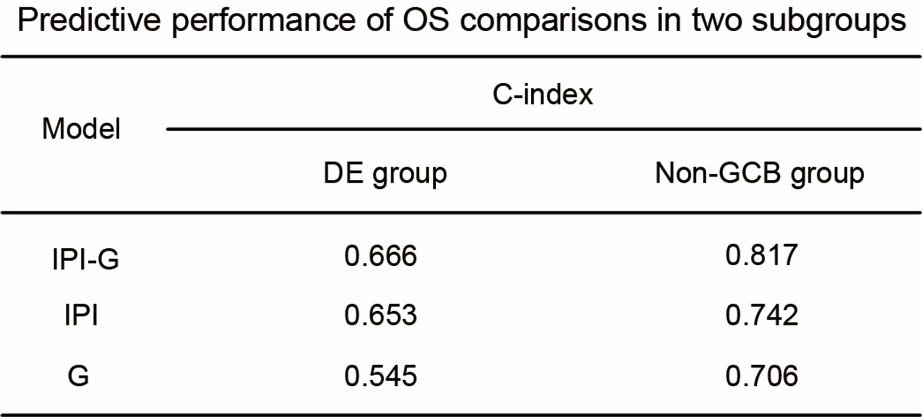
**

**Figure S5.** Predictive performance of OS comparisons in three models in two subgroups in the training PKUCH cohort, including DE subgroup and non-GCB subgroup. OS, overall survival; C-index, concordance index; IPI, International Prognostic Index; ­­G, genetic subtype; IPI-G, genetic subtype-based IPI; DE, BCL2/MYC double expressors; GCB, germinal center B-cell like.

**
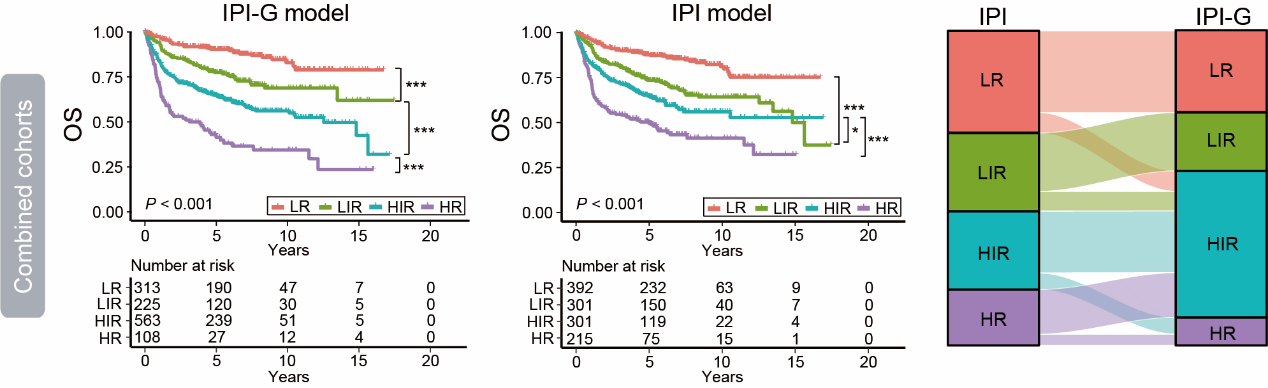
**

**Figure S6.** Kaplan-Meier survival curves and Sankey plot based on four-category integrated IPI-G prognostic model, compared with IPI model, in the combined 4 cohorts, including PKUCH cohort, NCI cohort, BCA cohort, and DHP cohort. *P* < 0.05 was considered statistically significant (*); *P* < 0.01 was considered highly statistically significant (**); *P* < 0.001 was considered extremely statistically significant (***). OS, overall survival; IPI, International Prognostic Index; IPI-G, genetic subtype-based IPI; LR, low risk; LIR, low-intermediate risk; HIR, high-intermediate risk; HR, high risk.

**
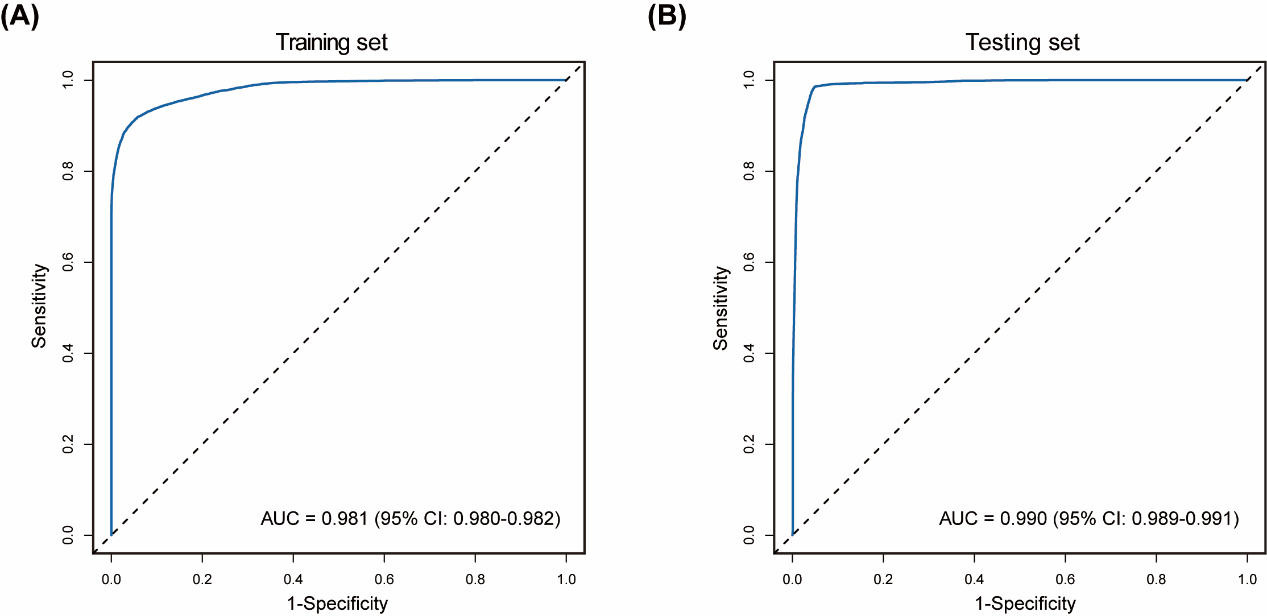
**

**Figure S7.** ROC of SomaticFinder in the training set (A) and testing set (B). Receiver operating curve; AUC, area under curve; CI, confidence interval.

**Table S1. Patient baseline clinical characteristics in the training PKUCH cohort**

| **Baseline characteristics** | **N (%)** |
| --- | --- |
| Age (years) |  |
| ≤ 60 | 58 (58.0%) |
| > 60 | 42 (42.0%) |
| Gender |  |
| Male | 61 (61.0%) |
| Female | 39 (39.0%) |
| Origin |  |
| Lymphatic node | 41 (41.0%) |
| Testis | 32 (32.0%) |
| Breast | 11 (11.0%) |
| Other | 16 (16.0%) |
| Hans COO classification |  |
| GCB | 27 (27.0%) |
| Non-GCB | 72 (72.0%) |
| NA | 1 (1.0%) |
| LDH |  |
| Normal | 52 (52.0%) |
| Elevated | 48 (48.0%) |
| ECOG PS |  |
| 0-1 | 96 (96.0%) |
| 2-4 | 4 (4.0%) |
| Ann Arbor stage |  |
| I-II | 51 (51.0%) |
| III-IV | 49 (49.0%) |
| Extranodal involvement (site) |  |
| 0-1 | 77 (77.0%) |
| ≥ 2 | 23 (23.0%) |
| IPI score |  |
| 0-1 | 46 (46.0%) |
| 2 | 26 (26.0%) |
| 3 | 20 (20.0%) |
| 4-5 | 8 (8.0%) |
| First-line treatment |  |
| R-CHOP | 97 (97.0%) |
| R-miniCCOP | 2 (2.0%) |
| R-CCOP | 1 (1.0%) |
| Response to treatment |  |
| CR | 82 (82.0%) |
| PR | 5 (5.0%) |
| PD | 13 (13.0%) |

Abbreviations: GCB, germinal center B-cell; NA, not appliable; LDH, lactate dehydrogenase; ECOG, Eastern Cooperative Oncology Group; PS, performance status; IPI, International Prognostic Index; COO, cell-of-origin; GCB, germinal center B-cell like; R-CHOP, rituximab, cyclophosphamide, doxorubicin, vincristine, and prednisone; R-CCOP, rituximab, cyclophosphamide, liposomal doxorubicin, vincristine, and prednisone; CR, complete remission; PR, partial remission; PD, progressive disease.

**Table S2. Targeted 74-gene panel for simplified LymphType algorithm**

| **Gene** | **Gene ID** | **Transcript** | **Typing characteristics** |
| --- | --- | --- | --- |
| *ACTB* | 60 | NM_001101 | Mutation |
| *ACTG1* | 71 | NM_001199954 | Mutation |
| *ARID1A* | 8289 | NM_006015 | Mutation + CNV |
| *BCL2L1* | 598 | NM_001317921 | Mutation |
| *BTG1* | 694 | NM_001731 | Mutation + CNV |
| *BTG2* | 7832 | NM_006763 | Mutation |
| *CD58* | 965 | NM_001779 | Mutation + CNV |
| *CD70* | 970 | NM_001330332 | Mutation |
| *CD79B* | 974 | NM_001039933 | Mutation |
| *CD83* | 9308 | NM_001251901 | Mutation |
| *CDKN2A* | 1029 | NM_000077 | Mutation + CNV |
| *CIITA* | 4261 | NR_104444 | Mutation + CNV |
| *CREBBP* | 1387 | NM_004380 | Mutation |
| *DTX1* | 1840 | NM_004416 | Mutation |
| *DUSP2* | 1844 | NM_004418 | Mutation |
| *DYSF* | 8291 | NM_001130981 | Mutation + CNV |
| *EIF4A2* | 1974 | NM_001967 | Mutation + CNV |
| *EP300* | 2033 | NM_001429 | Mutation + CNV |
| *ETS1* | 2113 | NM_001143820 | Mutation |
| *ETV6* | 2120 | NM_001987 | Mutation |
| *EZH2* | 2146 | NM_001203247 | Mutation |
| *FOXC1* | 2296 | NM_001453 | Mutation |
| *GRHPR* | 9380 | NM_012203 | Mutation |
| *HIST1H1B* | 3009 | NM_005322 | Mutation |
| *HIST1H1D* | 3007 | NM_005320 | Mutation |
| *HIST1H2BC* | 8347 | NM_003526 | Mutation |
| *HLA-A* | 3105 | NM_002116 | Mutation |
| *HLA-B* | 3106 | NM_005514 | Mutation + CNV |
| *ID3* | 3399 | NM_002167 | Mutation |
| *INTS1* | 26173 | NM_001080453 | Mutation + CNV |
| *IRF4* | 3662 | NM_001195286 | Mutation |
| *IRF8* | 3394 | NM_002163 | Mutation + CNV |
| *ITPKB* | 3707 | NM_002221 | Mutation |
| *JUNB* | 3726 | NM_002229 | Mutation |
| *KLF2* | 10365 | NM_016270 | Mutation |
| *KLHL14* | 57565 | NM_020805 | Mutation |
| *KMT2D* | 8085 | NM_003482 | Mutation |
| *MEF2B* | 100271849 | NM_001145785 | Mutation |
| *MPEG1* | 219972 | NM_001039396 | Mutation |
| *MYD88* | 4615 | NM_001172566 | Mutation |
| *NFKBIA* | 4792 | NM_020529 | Mutation |
| *NOL9* | 79707 | NM_024654 | Mutation |
| *NOTCH1* | 4851 | NM_017617 | Mutation |
| *NOTCH2* | 4853 | NM_024408 | Mutation |
| *OSBPL10* | 114884 | NM_001174060 | Mutation |
| *PACRGL* | 133015 | NR_133933 | CNV |
| *PIM1* | 5292 | NM_002648 | Mutation |
| *PIM2* | 11040 | NM_006875 | Mutation |
| *PRDM1* | 639 | NM_001198 | Mutation |
| *PRDM15* | 63977 | NM_001040424 | Mutation + CNV |
| *PRKDC* | 5591 | NM_001081640 | Mutation + CNV |
| *PRRC2A* | 7916 | NM_004638 | Mutation |
| *PRRC2C* | 23215 | NM_015172 | Mutation |
| *RFTN1* | 23180 | NM_015150 | Mutation |
| *RYR3* | 6263 | NM_001036 | CNV |
| *SETD1B* | 23067 | NM_015048 | Mutation + CNV |
| *SGK1* | 6446 | NM_001143676 | Mutation |
| *SOCS1* | 8651 | NM_003745 | Mutation |
| *SPEN* | 23013 | NM_015001 | Mutation |
| *SPIB* | 6689 | NM_003121 | CNV |
| *STAT3* | 6774 | NM_139276 | Mutation |
| *STAT6* | 6778 | NM_003153 | Mutation + CNV |
| *TBCC* | 6903 | NM_003192 | Mutation + CNV |
| *TBL1XR1* | 79718 | NM_001321193 | Mutation |
| *TET2* | 54790 | NM_001127208 | Mutation |
| *TMEM30A* | 55754 | NM_018247 | Mutation + CNV |
| *TNFAIP3* | 7128 | NM_006290 | Mutation |
| *TNFRSF14* | 8764 | NM_003820 | Mutation |
| *TNK2* | 10188 | NM_005781 | CNV |
| *TOX* | 9760 | NM_014729 | Mutation |
| *TP53* | 7157 | NM_000546 | Mutation + CNV |
| *WEE1* | 7465 | NM_003390 | Mutation |
| *ZFP36L1* | 677 | NM_001244701 | Mutation |
| *ZNF595* | 152687 | NM_001286054 | CNV |

Abbreviations: CNV, copy number variation.

**Table S3. Univariate Kaplan-Meier curve analysis of OS** **in the training PKUCH cohort**

| **Variables** | **HR** | ***P* value** |
| --- | --- | --- |
| age (> 60) | 4.22 | 0.003 |
| ECOG (2-4) | 5.64 | 0.002 |
| LDH (elevated) | 2.27 | 0.076 |
| Stage (III-IV) | 3.27 | 0.016 |
| Extranodal sites (≥ 2) | 1.98 | 0.156 |
| IPI | 4.16 | **0.004** |
| A53 | 2.69 | **0.168** |
| BN2 | 0.95 | 0.927 |
| EZB | 0.63 | 0.654 |
| MCD | 2.54 | **0.035** |
| ST2 | 0.00 | 0.235 |

Abbreviations: OS, overall survival; HR, hazard ratio; ECOG, Eastern Cooperative Oncology Group; LDH, lactate dehydrogenase; IPI, International Prognostic Index.

**Table S4. Comparisons of clinical features between the training cohort and validation cohort 1**

| **Variables** | **Training cohort**  **(PKUCH cohort)** | **Validation cohort 1**  **(NCI cohort)** | ***P* value** |
| --- | --- | --- | --- |
| Gender |  |  |  |
| Female | 39 (39.0%) | 82 (40.4%) | 0.816 |
| Age at diagnosis | 57 (26-89) | 61 (16-92) | 0.114 |
| IPI |  |  |  |
| IPI age (> 60) | 42 (42.0%) | 106 (52.2%) | 0.094 |
| IPI ECOG (2-4) | 4 (4.0%) | 48 (23.6%) | < 0.001 |
| IPI LDH (elevated) | 48 (48.0%) | 109 (53.7%) | 0.351 |
| IPI stage (III-IV) | 49 (49.0%) | 110 (54.2%) | 0.395 |
| IPI extranodal sites (≥ 2) | 23 (2.03%) | 33 (16.3%) | 0.155 |
| IPI score | 2 (0-5) | 2 (0-5) | 0.424 |
| 0-1 | 46 (46.0%) | 79 (38.9%) |  |
| 2 | 26 (26.0%) | 50 (24.6%) |  |
| 3 | 20 (20.0%) | 47 (23.2%) |  |
| 4-5 | 8 (8.0%) | 27 (13.3%) |  |
| COO classification |  |  |  |
| GCB | 27 (27.0%) | 93 (45.8%) | 0.002 |
| R-CHOP-like chemo | 100 (100.0%) | 203 (100.0%) | 1 |
| Time to last follow up (years) | 2.78 (0.32-9.13) | 4.49 (0.01-16.42) | < 0.001 |

Abbreviations: IPI, International Prognostic Index; ECOG, Eastern Cooperative Oncology Group; LDH, lactate dehydrogenase; COO, cell-of-origin; GCB, germinal center B-cell like; R-CHOP, rituximab, cyclophosphamide, doxorubicin, vincristine, and prednisone.

**Table S5. Comparisons of clinical features between the training cohort and validation cohort 2**

| **Variables** | **Training cohort**  **(PKUCH cohort)** | **Validation cohort 2**  **(BCA cohort)** | ***P* value** |
| --- | --- | --- | --- |
| Gender |  |  |  |
| Female | 39 (39.0%) | 117 (37.6%) | 0.805 |
| Age at diagnosis | 57 (26-89) | 64 (16-92) | < 0.001 |
| IPI |  |  |  |
| IPI age (> 60) | 42 (42.0%) | 183 (58.8%) | 0.003 |
| IPI ECOG (2-4) | 4 (4.0%) | 108 (34.7%) | < 0.001 |
| IPI LDH (elevated) | 48 (48.0%) | 163 (52.4%) | 0.443 |
| IPI stage (III-IV) | 49 (49.0%) | 166 (53.4%) | 0.446 |
| IPI extranodal sites (≥2) | 23 (2.03%) | 49 (15.8%) | 0.097 |
| IPI score | 2 (0-5) | 2 (0-5) | 0.016 |
| 0-1 | 46 (46.0%) | 105 (33.8%) |  |
| 2 | 26 (26.0%) | 90 (28.9%) |  |
| 3 | 20 (20.0%) | 53 (17%) |  |
| 4-5 | 8 (8.0%) | 63 (20.3%) |  |
| COO classification |  |  |  |
| GCB | 27 (27.0%) | 176 (56.6%) | < 0.001 |
| R-CHOP-like chemo | 100 (100.0%) | 311 (100%) | 1 |
| Time to last follow up (years) | 2.78 (0.32-9.13) | 7.87 (0.07-17.46) | < 0.001 |

Abbreviations: IPI, International Prognostic Index; ECOG, Eastern Cooperative Oncology Group; LDH, lactate dehydrogenase; COO, cell-of-origin; GCB, germinal center B-cell like; R-CHOP, rituximab, cyclophosphamide, doxorubicin, vincristine, and prednisone.

**Table S6. Comparisons of clinical features between the training cohort and validation cohort 3**

| **Variables** | **Training cohort**  **(PKUCH cohort)** | **Validation cohort 3**  **(DHP cohort)** | ***P* value** |
| --- | --- | --- | --- |
| IPI |  |  |  |
| IPI score | 2 (0-5) | 3 (0-5) | < 0.001 |
| 0-1 | 46 (46.0%) | 162 (27.2%) |  |
| 2 | 26 (26.0%) | 135 (22.7%) |  |
| 3 | 20 (20.0%) | 181 (30.4%) |  |
| 4-5 | 8 (8.0%) | 117 (19.7%) |  |
| COO classification |  |  |  |
| GCB | 27 (27.0%) | 256 (48.9%) | < 0.001 |
| R-CHOP-like chemo | 100 (100.0%) | 595 (100.0%) | 1 |
| Time to last follow up (years) | 2.78 (0.32-9.13) | 4.85 (0.02-11.38) | < 0.001 |

Abbreviations: IPI, International Prognostic Index; COO, cell-of-origin; GCB, germinal center B-cell like; R-CHOP, rituximab, cyclophosphamide, doxorubicin, vincristine, and prednisone.
